# A Cautionary Tale: Undetected H-type Tracheoesophageal Fistula in an Adolescent Male

**DOI:** 10.7759/cureus.57647

**Published:** 2024-04-05

**Authors:** Piotr R Więckowski, Joanna M Łysak, Ignacy Z Maciejewski, Marek Wolski

**Affiliations:** 1 Department of Pediatric Surgery and Organ Transplantation, Children's Memorial Health Institute, Warszawa, POL; 2 Department of Pediatric Surgery, Medical University of Warsaw, Warszawa, POL

**Keywords:** tracheoesophageal fistula, video assisted thoracoscopic surgery, persistent cough, tef, h-type tef, h-type fistula

## Abstract

An H-type tracheoesophageal fistula is a rare congenital anomaly consisting of an abnormal passageway between the esophagus and the trachea without the presence of esophageal atresia. This condition is usually detected early in infancy; however, some patients may receive a delayed diagnosis. Symptoms experienced by people affected with an H-type tracheoesophageal fistula vary greatly and may consist of bouts of coughing when swallowing liquids and recurring lower respiratory infections. The most commonly used initial diagnostic tests can produce falsely negative results. The treatment of choice for the majority of H-type tracheoesophageal fistulas is an open surgical procedure; however, the thoracoscopic approach has proven effective in cases where the fistula is located below the thoracic outlet. In this case report, we describe a patient whose diagnosis of H-type tracheoesophageal fistula was delayed by 13 years and who was successfully treated using thoracoscopic surgery.

## Introduction

Tracheoesophageal fistula (TEF), an abnormal connection between the esophagus and the trachea, is a common congenital abnormality affecting 4% of patients with tracheoesophageal malformations. A congenital H-type tracheoesophageal fistula is a rare (3.8% of all TEF cases) type of congenital malformation in which the esophagus is not obstructed [1]. However, an abnormal connection between those structures enables the passage of solids and liquids from the esophagus to the airways. Early diagnosis of H-type TEF may be difficult, commonly resulting in misdiagnosis. We describe a case of an adolescent male with tracheoesophageal fistula, which remained undetected for 13 years.

This article was previously presented as an abstract at the surgical case report session at the 18th Warsaw International Medical Congress in April 2023.

## Case presentation

Prior medical history

The patient was a 13-year-old Caucasian male with a history of congenital pneumonia and recurrent bronchitis. The mother of the patient reported observing a persistent dry cough, which began in the neonatal period. The patient received a diagnosis of asthma in early childhood. The tracheoesophageal fistula was excluded in infancy, but the records of diagnostic tests performed were not provided. Cystic fibrosis was excluded by performing sweat chloride level tests.

Differential diagnosis

The patient was admitted to the hospital with suspected interstitial lung disease and immunodeficiency. The patient complained of coughing, recurring respiratory infections, exercise-induced dyspnoea, and wheezing. A CT scan performed in a different facility showed suspected post-inflammatory nodules in the right lung and ground-glass opacity in the left lung. The thoracic esophagus was dilated and the entire length of the esophagus was filled with air. At the level of the third thoracic vertebra, the posterior wall of the trachea showed an uneven silhouette and suspected frothy discharge. The documentation provided consisted only of the CT scan description; the patient was unable to provide the CT scan images.

**Figure 1:**
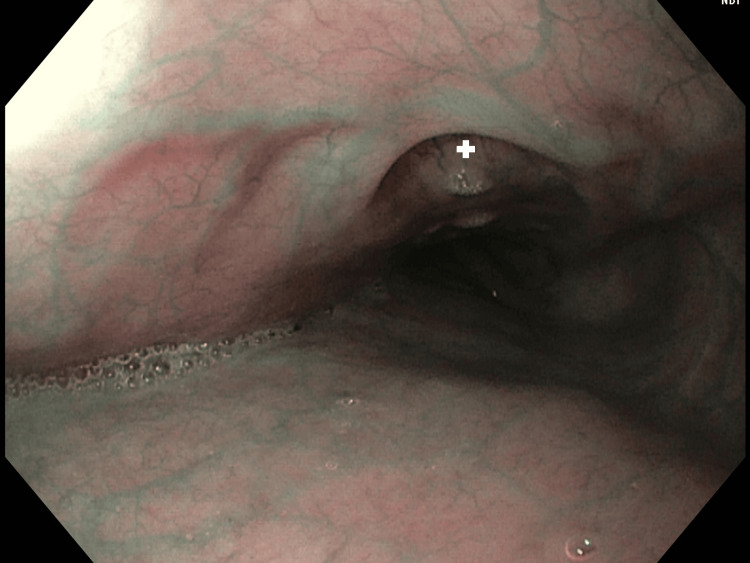
Gastroscopy performed during the diagnostic process for gastroesophageal reflux disease A plus (+) symbol is placed in the opening of the fistula into the esophagus.

Gastroesophageal reflux disease was suspected. Histopathology of the stomach and esophagus mucosa showed no abnormalities. Esophageal manometry was performed and no signs of achalasia were found. Gastroscopy concluded that a tracheoesophageal fistula was present, and Figure 1 shows the fistula opening into the esophagus.

The patient was diagnosed with an H-type tracheoesophageal fistula.

Surgical procedure

The patient returned to the hospital four months later to undergo surgical closing of the tracheoesophageal fistula.

**Figure 2:**
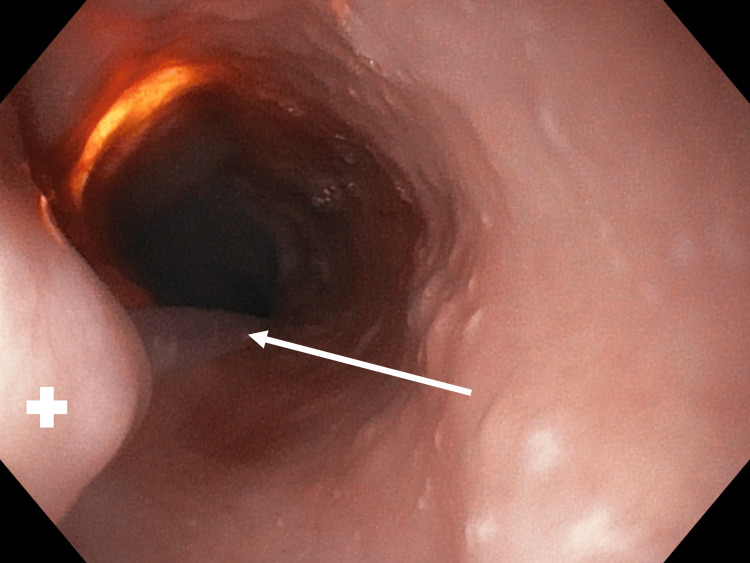
Gastroscopy performed after the insertion of the Nelaton catheter through the fistula during perioperative bronchoscopy A plus (+) symbol is placed on the opening of the fistula into the esophagus. The white arrow shows the Nelaton catheter inserted during the bronchoscopy.

A bronchoscopy was performed prior to the surgery. The location of the tracheoesophageal fistula was confirmed and described as “roughly 5 cm over the carina”. A vascular guidewire was inserted through the fistula and a Nelaton catheter was passed over the guidewire and through the fistula. Subsequently performed gastroscopy confirmed the catheter’s tip was present in the esophagus, which is shown in Figure 2. The patient was intubated and handed over to the surgical team.

**Figure 3:**
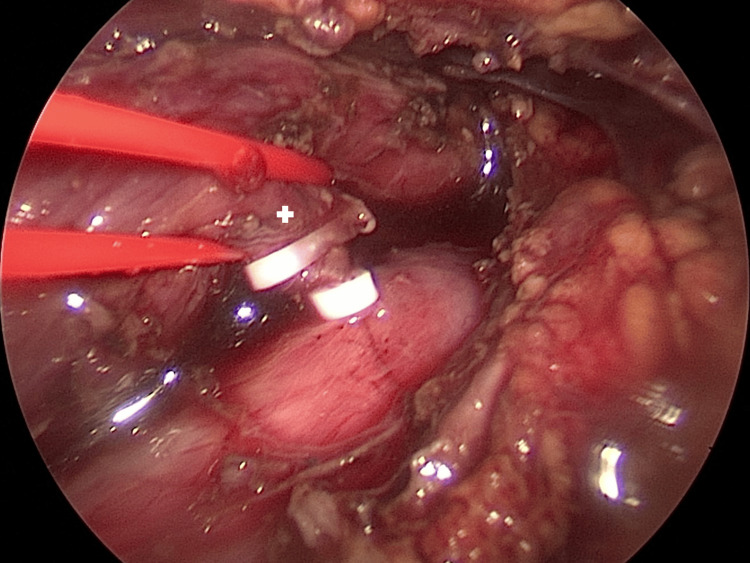
Thoracoscopic closing of the fistula. Two of the three clips are already applied to the fistula. A plus (+) symbol is placed on the fistula.

Thoracoscopic surgery was performed. The patient was placed on the left side with the right shoulder at a 30-degree angle. Two 5 mm trocars were inserted, one in the mid-axillary line and one in the posterior axillary line. The 10 mm trocar was inserted near the spine. The region of the fistula was isolated by exposing the esophagus and trachea near the upper thoracic opening. The fistula was dissected, suspended on a rubber band, and ligated using three clips. Figure 3 shows two of the three clips applied to the fistula. Air was suctioned from the thoracic cavity, and the thoracic wall was closed.

The postoperative period was uneventful. The patient received a mushy diet for one day and was instructed to avoid exertion and change the wound dressings regularly.

A follow-up visit was performed after three months, during which the patient denied any symptoms. The physical examination performed during the follow-up did not produce any findings.

## Discussion

The esophagus and the trachea are two separate, hollow organs with varying functions that share a common embryological origin - the caudal foregut. Given this, congenital abnormalities affecting both the esophagus and trachea may arise from the improper septation of the caudal foregut between the fourth and fifth weeks of gestation [1]. The H-type tracheoesophageal fistula is an abnormal duct connecting the trachea with the esophagus in the absence of esophageal atresia. In this case, as the continuity of the trachea and esophagus was preserved, the patient was asymptomatic until the introduction of feeding and the symptoms were intermittent and uncharacteristic [2,3].

The most common variant of the tracheoesophageal fistula is the distal tracheoesophageal fistula. In this case, the distal esophagus is connected to the trachea and the proximal esophagus ends in a blind pouch. Neonates with this condition usually experience symptoms immediately from birth [4]. Diagnosis is made after the unsuccessful passage of a nasogastric or orogastric catheter, with the AP chest radiogram confirming the presence of a curled-up catheter in the esophageal pouch. There is no such convenient test to diagnose an H-type tracheoesophageal fistula. The standard diagnostic test is a pull-back tube esophagogram, during which a tube is inserted into the distal esophagus and liquid contrast material is infused through the tube. The tube is then pulled cephalad and the infusion is repeated. The presence of a fistula is confirmed when the contrast medium leaks into the trachea on the radiogram [3]. A proposed alternative to this test is a contrast media swallow test [5]. However, because of our experience of false negatives occurring during the swallow test and pull-back esophagogram, we choose to perform bronchoscopy or gastroscopy with the passage of the catheter through a suspected fistula to obtain a definite diagnosis of the H-type tracheoesophageal fistula. We suspect that our patient had a contrast media swallow test performed, the negative result of which resulted in the described exclusion of the tracheoesophageal fistula in the provided documentation. The precise location of the fistula can be visualized by passing some kind of catheter or guide wire through the fistula during a perioperative bronchoscopy [6-8]. It improves the visibility of the fistula during the surgery, enabling safer and more precise dissection.

Infants with H-type TEF may present with coughing, choking, salivation, vomiting, and cyanosis induced by the onset of feeding [2-4]. Adults affected by a previously undiagnosed congenital tracheoesophageal fistula present with persistent cough and recurrent respiratory infections [3,9-12]. Bouts of coughing when swallowing liquids are considered to be pathognomonic for this condition [11]. The severity of these symptoms is highly variable among affected individuals [3,6,9-12].

The treatment of choice is an open surgical procedure, either through a cervical or a thoracic approach. The choice between the cervical and thoracic approaches is determined by the location of the fistula relative to the vertebrae. The thoracic approach used in this case is reserved for fistulae located below the second thoracic vertebra [13], although some authors contest this criterion and suggest the cervical approach even in cases of the fistula occurring below T2 [14]. It should be noted that those divisions were established in infants whose anatomic relations are vastly different from the anatomic relations in an adolescent male. Thoracoscopic surgery is considered less invasive and has been used to repair H-type fistulas as well [7-10,13,15-19]. Parolini et al., in a 2014 systematic review, concluded that the thoracoscopic approach is a safe and effective technique, avoiding the scarring and morbidity associated with thoracotomy; however, it should be considered that the evidence is limited to retrospective case series and case reports [13]. Toczewski et al. described, in their 2020 case series, probably the most extensive review of thoracoscopic repairs of the TEF [8]. Their experience proves that the thoracoscopic approach to this surgery is a viable and safe method; however, some aspects of the surgery, such as the necessity of transection of the fistula or the passage of the guidewire prior to the procedure, have not been studied and compared, as the authors stated [8]. Reports of thoracoscopic repair of an H-type tracheoesophageal fistula in adults are extremely limited; we were able to find only three such works, which all concluded that the thoracoscopic approach to this procedure is possible and curative [9,10,19].

In the case of our patient, as the fistula was on the level of the third thoracic vertebra, an approach from the thoracic cavity seemed the most optimal. The experience of this team, led by Marek Wolski, facilitated the less invasive thoracoscopic approach. This choice resulted in an uncomplicated perioperative period and an excellent long-term outcome as described above.

## Conclusions

This case is an important cautionary tale. Compared to other types of TEF, an H-type tracheoesophageal fistula is easy to miss considering its indistinct clinical manifestation. Recurring pneumonia and respiratory tract infections in children, although usually not linked to such conditions, should inspire doctors to consider an H-type TEF in their diagnostic process. The contrast media swallow test should not be used to exclude the presence of the tracheoesophageal fistula, as it produces false negatives that can delay diagnosis by many years, which was the main cause of the delayed diagnosis of our patient. In the presence of an experienced team, thoracoscopic surgery should be considered as the less invasive approach to treat H-type tracheoesophageal fistulas.
